# Antifungal Microbial Agents for Food Biopreservation—A Review

**DOI:** 10.3390/microorganisms5030037

**Published:** 2017-07-08

**Authors:** Marcia Leyva Salas, Jérôme Mounier, Florence Valence, Monika Coton, Anne Thierry, Emmanuel Coton

**Affiliations:** 1 Laboratoire Universitaire de Biodiversité et Ecologie Microbienne (LUBEM EA3882), Université de Brest, Technopole Brest-Iroise, 29280 Plouzané, France; marcia.leyvasalas@univ-brest.fr (M.L.S.); jerome.mounier@univ-brest.fr (J.M.); monika.coton@univ-brest.fr (M.C.); 2 UMR1253 Science et Technologie du Lait et de l’Œuf, INRA, Agrocampus Ouest, 65 rue de Saint Brieuc, 35000 Rennes, France; florence.valence-bertel@inra.fr (F.V.); anne.thierry@inra.fr (A.T.)

**Keywords:** antifungal, bioprotection, biocontrol, food, post-harvest, fungi, lactic acid bacteria, propionibacteria, *Bacillus*, fermentates, molecules

## Abstract

Food spoilage is a major issue for the food industry, leading to food waste, substantial economic losses for manufacturers and consumers, and a negative impact on brand names. Among causes, fungal contamination can be encountered at various stages of the food chain (e.g., post-harvest, during processing or storage). Fungal development leads to food sensory defects varying from visual deterioration to noticeable odor, flavor, or texture changes but can also have negative health impacts via mycotoxin production by some molds. In order to avoid microbial spoilage and thus extend product shelf life, different treatments—including fungicides and chemical preservatives—are used. In parallel, public authorities encourage the food industry to limit the use of these chemical compounds and develop natural methods for food preservation. This is accompanied by a strong societal demand for ‘clean label’ food products, as consumers are looking for more natural, less severely processed and safer products. In this context, microbial agents corresponding to bioprotective cultures, fermentates, culture-free supernatant or purified molecules, exhibiting antifungal activities represent a growing interest as an alternative to chemical preservation. This review presents the main fungal spoilers encountered in food products, the antifungal microorganisms tested for food bioprotection, and their mechanisms of action. A focus is made in particular on the recent in situ studies and the constraints associated with the use of antifungal microbial agents for food biopreservation.

## 1. Introduction

Today, food losses are a major concern worldwide especially with an ever-growing world population and the fact that approximately one-third of all food produced for human consumption is either lost or wasted [1]. Each year, an estimated 1.3 billion tons of food are lost or wasted as stated by the Food and Agriculture Organization of the United Nations [2]. When considering each food category, these losses correspond to 40–50% of root crops, fruits, and vegetables; 35% of fish and seafood; 30% of cereals; and 20% of meat, oil seed, and dairy products [2]. In developing countries, post-harvest loss rates are high with 30–40% occurring during the post-harvest and processing stage [3], while in industrialized countries similar loss percentages (30%) occur at the retail or consumer levels [1]. The reasons for this massive global food loss are diverse, but microbial spoilage, which affects organoleptic product quality (aspect, texture, taste, and aroma), plays a major role. Among spoilage microorganisms, fungi are a major issue at any stage of the food chain because of their ability to grow in different and even harsh environments [4]. Beyond their negative impact on food quality, some fungal genera such as *Aspergillus*, *Penicillium*, *Alternaria*, and *Fusarium* have the ability to produce secondary metabolites that can have a toxic effect on humans and animals and are therefore named mycotoxins. Moreover, mycotoxins are able to withstand various food processing steps and can thus lead to food safety concerns [5]. Fungi, mainly under an airborne spore form (either sexual or asexual), can settle and grow at different stages of a product life, i.e., at the field, post-harvest, during processing, storage and handling steps by the producer, wholesaler, retailer, and consumer. The impact of contamination at each level can obviously lead to economic losses at both the producer and consumer levels [1]. Moreover, producer losses can be reinforced by a negative brand image induced by consumer dissatisfaction.

In this context, it is crucial to reduce food losses by controlling fungal contamination at all stages of food process chains. Three main stages can be defined to group fungal contamination factors: (i) the field, where water, soil, and air are natural fungal niches; (ii) raw materials—such as post-harvest crops, meats, and milk—where fungal occurrence is related to food management during harvest or collecting, transportation, storage, and packaging [4] and (iii) during food processing while manufacturing dairy, bakery, dry-ripened, and drink products. For field crops, fungal contamination is usually controlled by using synthetic fungicides associated with some crop management practices such as crop rotation, use of resistant cultivars, and tillage [6]. Concerning raw materials, fungicides are widely applied to protect post-harvest fruits and vegetables, but other treatments such as disinfection with ozone, chlorine, acidified hydrogen peroxide, pH change with sodium bicarbonate, surface sterilization using irradiation or thermal treatments, as well as waxing with active coatings containing fungicidal agents and preservatives are also used. Moreover, different packaging techniques are also used to protect crops from mechanical damage, which is the most common entry point of microbial infections [7,8]. During food processing and raw material storage, good hygiene practices, a hazard analysis critical control point (HACCP) system, efficient equipment and air decontamination, and control of air pressurization to prevent air flow from the dirtiest to cleanest areas—cleanroom technologies (aseptic or ultraclean technologies)—can also be applied to prevent contaminations. Moreover, hurdle technologies are also applied according to the given process and raw materials. They can include thermal treatments such as cooling or heating by pasteurization, sterilization, and heating at ultra-high temperatures (UHT); water removal by drying, freeze-drying, smoking; water activity reduction by addition of high sugar and salt concentrations; pH modification by immersion in vinegar or other acids; and the use of non-thermal preservation such as modified atmosphere packaging to reduce O_2_ and increase CO_2_ levels; and finally high-pressure treatment and high electric field pulse to control or inactivate microorganisms and spores especially in foods and beverages such as yogurts, soups, sauces, liquid eggs, fruit and vegetable juices, raw milk and whey, soft drinks, and alcoholic beverages [9,10]. Moreover, chemical preservatives—including benzoate, propionate, sorbate, nitrate, nitrite, and sulfites—are used to avoid fungal spoilage [11]. The only microbial derived antifungal compound used as food preservative is natamycin (E235). It is produced by *Streptomyces nataliensis* and belongs to the group of polyene macrolide antimycotics. The use of natamycin on cheese as a surface treatment is approved by most countries and its addition into other foods depends on legislation [12].

The potential adverse environmental and health effects of certain fungicides and preservatives have led to more natural methods. Concerning crops, undesirable concentrations of residual pesticides on harvested products, regulations, acquired resistances by fungi, and environmental impact have led to biocontrol agent development (reviewed for fruits and vegetables by [13] and for cereals by [14] currently a fast-growing sector, is also in need of natural solutions to reduce crop losses. Several products composed of bacteria, fungi, and yeasts are currently commercialized worldwide [15,16,17,18]. Concerning raw materials and processed foods, preservatives are widely used. However, while their use is regulated in many countries, ingestion of some of them raises questions about their potential adverse health effects, especially when considering their chronic consumption over years [11]. Moreover, fungal spoiler resistances have been previously reported in some cases [19,20]. In this context, there is a strong societal demand, supported by public authorities, for less processed and preservative-free foods, such as ‘clean label’ products, and the use of natural alternatives. Such alternatives mainly correspond to the use of essential oils [21,22,23] and antagonistic microorganisms as preservation tools [24,25,26,27,28,29]. The use of natural or added microorganisms, fermentates (i.e., an ingredient produced by the fermentation of a variety of raw materials by a microorganism), or their metabolites to extend food shelf-life and/or increase food safety (through antibiosis of pathogenic or spoilage microorganism) is referred to as ‘biopreservation’ or ‘biocontrol’ [30]. This alternative approach is complementary to hurdle technologies. Biopreservation by lactic acid bacteria (LAB) is currently the principal alternative to preservatives in food and is widely studied because of the role and long history of use of LAB in fermented foods, their ability to produce antifungal metabolites, and their GRAS (Generally Regarded as Safe) and Qualified Presumption of Safety (QPS) status in the USA and EU, respectively. The use of antifungal LAB for food biopreservation was previously reviewed in 2013 by Crowley [24]. Other antifungal microorganisms to prevent post-harvest fungal spoilage has been reviewed by Sharma et al. (2009) and Liu et al. (2013) [29,31]. Since then, there have been significant advances in the knowledge surrounding the use of antifungal microorganisms. Therefore, the objective of this review is to report the advances on LAB applications as antifungal agents (over the last three years) as well as other antifungal microorganisms, namely non-LAB bacteria, yeasts, and molds that can be used at the post-harvest stage, for raw materials, or during food processing. Crop biocontrol will not be considered in this review and the reader is referred to the recent review for cereals by Oliveira et al. 2014 [14], Punja & Utkhede, 2003 [32], Milind et al. 2016 [33], and Nguyen et al. 2017 [34]. It will detail the main fungal spoilers encountered in different raw or processed foods, antifungal microorganisms that can be used for food bioprotection, and current knowledge about their mechanisms of action. Finally, advances and constraints associated with the use of antifungal biopreservatives in food and the future areas of research required in this field will be discussed.

## 2. Fungal Spoilers

### 2.1. Quality and Safety Issues

Food products are susceptible to fungal spoilage due to their natural nutrient rich composition. This susceptibility does however depend on various factors, namely (i) the food matrix nature (living material or not, liquid or solid), its composition (nutrient content, solute type), and biological (e.g., its natural microbiota), physical, and chemical parameters (water activity, pH); (ii) management during harvesting of fruits and vegetables (maturity, handling) and raw material storage (temperature, hygrometry, and duration); (iii) technological processes applied during manufacture (e.g., heating, drying, salting, fermentation, preservative addition) including cleaning/disinfection steps; and (iv) storage conditions after manufacturing (type, atmosphere and extent of packaging, temperature, relative humidity). Food matrix intrinsic characteristics and also associated extrinsic factors will control the development of certain fungal genera or species. This usually leads to the selection or dominance of one or more fungal species on a given matrix.

Regarding quality, the impact of fungal contaminations can lead to visual and/or other defects. Regarding visual defects, the most obvious is product aspect with apparent fungal growth due to noticeable presence of thallus or yeast colonies that eventually leads to rotting. The presence of black, white, green, pink, or yellow spots can also be associated with fungal development [4]. This usually leads to elimination of the entire product at the industrial or consumer level. According to the nature of the commodity and the contaminant type, the impact might be less evident to observe, but fungal metabolism can lead to various organoleptic defects including gas production, off-flavors, and texture changes. One of the best known off-flavors associated with fungal contamination corresponds to animal-like aroma development linked to volatile phenols (4-ethyl phenol, guaiacol, or catechol) in wine and cider by *Brettanomyces* spp. [35,36]. In wine, molds have also been shown to be responsible for the production of earthy/moldy (e.g., geosmin, methylisoborneol) off-flavors [37,38]. Gas production have also been observed. For example, the presence of bubbles was observed in packaged meat inoculated with *Kazachstania psychrophila* [39]. *Kazachstania servazzii* has been associated with severe package swelling of prepared fresh pizzas [40] while yogurt has been shown to be contaminated by fermentative yeasts (e.g., *Meyerozyma guilliermondii)* that led to top lid bulging [41]. Moreover, due to enzymatic lipolytic, proteolytic, and amylolytic activities, product texture can also be affected. For example, *Yarrowia lipolytica* can produce off-flavors, stimulate the formation of biogenic amines, and negatively affect cheese texture [42].

Regarding safety concerns, fungal presence and growth may also lead to health hazards associated with the presence of mycotoxins that can be produced by species belonging to the *Aspergillus, Penicillium*, *Fusarium*, and *Alternaria* genera. This aspect is of importance as about 25% of raw materials produced by agriculture worldwide are estimated to be contaminated by fungi and mycotoxins [43,44]. Some mycotoxins can induce toxic, carcinogenic, and mutagenic reactions in humans at low concentrations [5]; aflatoxins, ochratoxin A, patulin, fumonisins, zearalenone, T-2 and HT-2 toxins, and deoxynivalenol are considered to be the most significant mycotoxins. The main problem is the presence of mycotoxins in processed foods as the presence of the mycotoxigenic spoilers can no longer be detected, but these metabolites are usually resistant to technological treatments. This has led to regulations on mycotoxin limits in various countries [45]. Among important mycotoxins in food spoilage, aflatoxins and ochratoxin A are produced by several members of *Aspergillus* while OTA can also be produced by some *Penicillium* species. *Aspergillus flavus,* the main aflatoxin producer, is a common spoiler of various commodities (fruits and vegetables, spices, cereals, bread, and nuts such as peanuts, almonds and pistachios). Fumonisins are produced by *Fusarium* and *Aspergillus* species. For example, *Aspergillus awamori* strains infecting onions and *Aspergillus niger* found in coffee beans and grapes produced fumonisins and ochratoxins. Moreover, a further potential risk is associated with products derived from raw materials containing these mycotoxins [5]. Patulin, which is non-carcinogenic but immunotoxic and neurotoxic in animals, can be produced by several *Alternaria*, *Paelomyces*, *Aspergillus*, and *Penicillium* species. For example, patulin can be produced by *Penicillium expansum,* a common apple spoiler, and then be transferred to juices and other derived products (e.g., baby food, apple sauce) [5]. Fungi from the *Alternaria* genus are common on post-harvest fruit decay. They can produce different mycotoxins such as alternariol, alternariol monomethyl ether, altenuene, altertoxins I, II, III (ATX-I, -II, -III), and tenuazonic acid that have a mutagenic effect [46]. So far, the mycotoxin risk is considered for a single mycotoxin, however, mycotoxin contamination rather corresponds to multi-occurrence due to the simultaneous presence of several mycotoxigenic species and/or of species able to produce several mycotoxins [47].

### 2.2. Isolation and Identification of Fungal Spoilers

Before developing biopreservation or biocontrol tools (i.e., selecting antifungal microbial agents) to control fungal spoilers, it is fundamental to determine the main fungal contaminants and their occurrence in a given food. This mainly relies on their isolation and accurate identification at the species level, which will consequently also provide information on their mycotoxigenic potential. In the context of fungal spoilage, contamination of a given food product is usually linked to a limited number of species and, therefore, culture-dependent methods are the most appropriate. The first step corresponds to isolation and/or enumeration of these microorganisms which can be performed not only from the contaminated food but also from other potential contamination sources like air and surfaces. The use of specific media is a classical step to isolate and determine total counts as well as morphotype observation. After isolation of the observed contaminant, identification is performed using dedicated methods. Although molds can be presumptively identified from their morphology (e.g., by Pitt test), molecular methods have become the gold standard for fungal identification. PCR amplification and sequencing of the D1/D2 domain of the large subunit of ribosomal RNA (26S) and of the internal transcribed spacer (ITS) are routinely performed for yeast and molds, respectively [48,49,50,51]. However, concerning molds, ITS is not always informative enough for some species, and other gene targets can be sequenced to complete identification at the species level. For example, the β-tubulin gene is commonly used for *Penicillium* and *Aspergillus* spp., actin gene for *Cladosporium* spp., and the elongation factor gene for *Fusarium* spp. It is more and more common to use a combination of taxonomically relevant genes to facilitate identification and potentially describe new spoiler agents.

It is worth noting that the constitution of fungal spoiler culture collections would be of great interest to study antifungal cultures. These collections are especially of interest if they contain various strains of a given species. Indeed, most studies only focus on one strain of a given fungal spoiler, raising questions about the representativeness of the selected target strain.

### 2.3. Fungal Spoilers and Food Chains

The diversity of fungi and yeast as food spoilers has been explored in different studies and food products. Notably, fungal species are not always detrimental for food production and some species are even crucial, especially in fermented foods to obtain the typical organoleptic traits of the final product, e.g., *Saccharomyces cerevisiae* in fermented beverages, *Penicillium camemberti* in mold-ripened cheeses, or *Aspergillus oryzae* in soy sauce. Moreover, it should be kept in mind that a fungal species that is expected and often deliberately added in some fermented foods can be a spoiler in another product. For example, *Penicillium roqueforti*—a species essential for blue-veined cheese production—can spoil grated cheese or fresh cheese.

When considering food spoilage by fungi, two main food categories have been proposed by Pitt & Hocking, namely (i) fresh or perishable foods (subcategorized in living and non-living cells) and (ii) stored or processed foods [4]. For a complete view of fungal spoilage of these products, one should refer to these authors’ books, which are considered as the reference in the domain. Post-harvest fungal spoilage of crops is most often referred to as ‘post-harvest disease’, and spoilers are referred to as ‘pathogens’, therefore these terms will also be used hereafter.

#### 2.3.1. Fresh or Perishable Foods

Concerning foods and raw materials of plant origin, fruits and vegetables are mainly susceptible to fungal contamination during the ripening stage because of changes in pH, skin, carbohydrates, and defenses that induce favorable conditions for fungal spoilers. Fungi induce visible symptoms on post-harvest crops including discoloration and tissue lesion formation. Their presence leads to spoilage of a variety of fruits including citrus, pome, berry, stone, tropical, and solanaceous fruits. Fungal pathogens of fruits and vegetables have been largely documented [5,8,52]. Tubers and other vegetables—including bulbs, crucifers, cucurbits, and legumes—are less affected than fruits by fungal pathogens, partly because of their pH making them a more suitable environment for bacterial pathogens [52]. Fungal species belonging to the *Penicillium*, *Botrytis*, *Monilinia*, *Rhizopus*, *Alternaria*, *Aspergillus*, *Fusarium*, *Geotrichum*, *Gloeosporium*, and *Mucor* genera are responsible for many of the most important postharvest diseases [29].

For foods and raw materials of animal origin, fresh milk and fish are less susceptible to fungal spoilage but highly affected by bacterial spoilage. Concerning meat, fungal spoilage can occur, especially during refrigeration, even if bacterial spoilage is predominant [53]. Despite a need for more studies, *Cladosporium*, *Penicillium*, and *Aureobasidium* species have been reported to provoke black spots, while *Thamnidium* spp. can cause ‘whiskers’ on carcasses. Yeast belonging to *Cryptococcus*, *Candida*, and *Yarrowia* genera have been reported on aerobic packaged food and can provoke off-flavors and aspect defects, such as slime or spots [54].

#### 2.3.2. Stored and Processed Foods

Stored and processed foods include a wide variety of food products, in which water activity (a_w_) determines viability and functionality of microrganisms. Most of them can not multiply below 0.900 a_w_, while xerophilic fungi can survive at 0.755–0.605 a_w_ [55]. Final moisture content and water activity are linked to the technological processes used (e.g., fermentation, drying, salting, evaporation, and freeze-drying) [10]. For intermediate and high water activity products, the main food categories concerned by fungal spoilage are dairy products (yogurt, cream, butter, cheese…). These products, usually kept refrigerated, can be affected by both yeasts, especially *Candida* spp., *Yarrowia lipolytica* and *M. guilliermondii,* and molds, mainly *Penicillium, Mucor* and *Cladosporium* spp. [49]. Processed meats (chilled meats, bacon), which exhibit an intermediate moisture content, are commonly spoiled by *Penicillium, Aerobasidium, Cladosporium*, and *Eurotium* spp., but also *Debaryomyces hansenii*, *Y. lipolytica* and *Candida* spp. For low water activity food products such as cereals, nuts, spices, dried milk, dried meats (dried beef, biltong) and, fermented meats (dry cured ham, salami, and fermented sausage) [4]. *Eurotium*, *Aspergillus,* and *Penicillium* spp. are the main spoilers. Spoiling yeasts such as *D. hansenii* and *Y. lipolytica* have also been found in dried meats [56] and *Candida* spp. in fermented meat [4]. Some of the most xerophilic species are *Wallemia sebi*, *Eurotium repens*, *Eurotium halophilicum*, *Xeromyces bisporus,* and the yeast *Zygosaccharomyces rouxii* found in concentrated foods (e.g., fruit concentrates, jams and confectionery), cereals and spices, [55,57,58]. Recently, *Aspergillus penicillioides* found in dried fish [59] was shown to be the most xerophilic fungal species registered to date as it was able to germinate at 0.585 a_w_ [60].

Heat treatments, especially when applied to acidic foods, usually allow for fungal destruction. However, ascospores of some species (*Byssochlamys nivea*) are resistant and can cause spoilage in fruit puree or fruit juices [61]. Noteworthy, contamination of heat-treated products can occur as post-contamination by non-heat-resistant species. This is the case of bakery products, with breads being particularly susceptible to fungal spoilage. The most frequently found genera are *Penicillium* [62] *Aspergillus, Wallemia,* and *Eurotium* [63]. In salted foods, *Wallemia sebi*, *Aspergillus* spp*.* and *Eurotium* spp. are the main spoilers associated with salted fish [64].

## 3. Antifungal Microorganisms in Food

As previously stated, to answer the strong societal demand for less processed and preservative-free foods, biopreservation has received growing interest for improving food quality and safety [14,65]. In past years, many strains from various microbial species that harbor antifungal properties have been identified. They have been isolated from various sources, such as fruits, vegetables, cereals, milk, meat, and other food-related products. The isolation of new bioprotective cultures has recently been extended to other environments, such as deep-sea [66] and Antarctic soil samples [67] in order to discover microorganisms potentially producing new antifungal metabolites.

Screening steps are required to find efficient antifungal microorganisms as antifungal activity levels and the spectrum of the inhibited fungal targets greatly vary depending on the considered species, and from strain to strain within a species. For example, up to 75% of variation was observed between five strains of *Lactobacillus casei* tested for their potential to inhibit the growth of four spoilage molds [68]. In another study, only a few *L. plantarum* isolates among 88 screened showed a wide spectrum of fungi inhibition [69]. In another example, among 55 yeast isolates (*Aureobasidium pullulans, Cryptococcus magnus, Hanseniaspora uvarum*, *Candida zeylanoides*, *Candida sake*, *Rhodotorula mucilaginosa*, and *Pseudozyma aphidis*) from the surface of grape varieties, 58% were able to inhibit *A. tubingensis* growth in vitro and 27 from 37 strains of *A. pollulans* were antifungal indicating a strain-dependent trait [70].

### 3.1. Screening and Validation Methods

#### 3.1.1. In Vitro Screening

Candidate bacteria, yeasts, and molds are generally first isolated from various biotopes and purified in order to be tested for their antifungal properties. These candidates are then tested against one or several fungal targets. As stated in the previous chapter, the choice of fungal target(s) is crucial, since they should be representative of the dominant species associated with a given food product and since the antifungal effects observed depend on the fungal species. For example, the inhibition of three berry contaminants—namely *Botrytis cinerea*, *Penicillium digitatum* and *Penicillium italicum*—by four antifungal yeasts varied according to the target [71]. Also, growth inhibition of seven targets from the *Penicillium, Aspergillus*, and *Cladosporium* genera varied for the 88 *Lactobacillus plantarum* strains tested [69].

Several approaches, ranging from classical methods in agar plates to high throughput assays in multi well-plates, have been developed to screen microorganisms for their ability to inhibit spoilage fungi. In the spot-on-lawn assay, cultures or cell-free supernatants of the tested strains are spotted over an agar plate, over which a second layer containing the fungal target is poured. In the agar diffusion method, the tested agent is spotted over an agar layer inoculated with the fungal target. In both these assays, formation of an inhibition halo after incubation around the spots reveals the antifungal activity. A dual culture technique has also been used to test the antagonistic effect of a potential antifungal mold against a target mold, in which agar plugs containing the target and the antifungal molds, respectively, were inoculated at the opposite sides over an agar plate [70,72]. Another screening approach corresponds to the investigation of the enzymatic activities required to degrade the cell wall of the fungal target as shown by Tokpah et al. (2016) that screened bacteria for control of a rice pathogen, *Magnaporthe grisea*. In this context, activity of cell wall degrading enzymes—such as cellulases, proteases, chitinases, and glucanases—was assessed by plating bacteria on agar media containing substrates of these enzymes where active bacteria induced clearing zones [73].

For high-throughput screening purposes, agar plate methods have been recently adapted in 24-well plates. The fungal target can be included in an agar medium and poured in each well over a first agar layer spotted with the potential antifungal strains [74]. Alternatively, spores of the target mold are inoculated on the surface of the first agar layer containing the potential antifungal strains [63].

Most in vitro screening experiments of LAB antifungal activities have been performed using synthetic media, such as the de Man, Rogosa, and Sharpe (MRS) agar medium. The composition of MRS can strongly impact the expression of antifungal activity by LAB because it contains acetate, which may reinforce LAB antifungal activity and artificially inflate the number of active isolates, as mentioned in several studies [63,75,76]. Similarly, the presence of several antifungal compounds—such as cyclic and linear peptides and diketopiperazines—has been reported in the classical lactate-tryptone-yeast extract medium used to grow propionibacteria [77]. Other important points are the nature and concentration of sugars and the buffering capacity of the medium, which are highly expected to influence the amount of organic acids and the final pH of the medium, thus modifying antifungal activity. Some alternatives to the use of synthetic laboratory media have been recently reported. Particular attention has been paid to design media more closely related to foods and usable in 24-well plates to screen bacterial antifungal activity. This approach was recently used with a wheat flour hydrolysate agar medium and with yogurt [63,78]. Similarly, a method using a model cheese distributed in 24-well plates was also recently developed allowing for high-throughput antifungal activity screening of either LAB fermentates or LAB starters [79].

Whatever the considered screening experiments, it is important to take into account the conditions prevailing in situ to develop relevant screening approaches. Actually, several studies have shown that strains exhibiting in vitro antifungal activity were far less or even no longer active when tested in the food products [63,80,81,82]. When grown on a wheat flour medium, 20% of the 270 tested LAB strains representing six genera screened were found to inhibit the five fungal targets tested while none were detected when grown in MRS agar without acetate [63].

For all these reasons, results of in vitro screening are highly heterogeneous in terms of proportion of strains and nature of species harboring antifungal activity. To maximize the chances of finding efficient strains in situ, approaches based on the use of food-related media and screening conditions should be further developed, in particular at a high throughput scale for large collections of strains.

#### 3.1.2. Validation by Challenge-Test in the Food Products

In all cases*,* in situ evaluation in the actual food products of the antifungal activity of the microorganisms or their metabolites selected from in vitro screening is essential, since, as stated above (see also chapter V), many studies report discrepancies between results observed in vitro and in situ [83].

The developed approaches depend on the type of foods to be protected (raw material or processed food, fermented or not), and on the antifungal microorganism used, to maximize efficiency without adverse effects on the sensory characteristics of the food or on the environment. Biopreservation/biocontrol can be achieved either (i) by adding an active ingredient constituted of a fraction containing purified metabolites, a cell-free supernatant (CFS), a fermentate; or (ii) by using cells expected to grow on or in the food product.

Concerning postharvest fruits, bacterial cells, yeast cells, and cell-free supernatants of molds were evaluated by spraying them onto the surface of an intact fruit or on a wound, before inoculation of the fungal target [31,84,85,86]. Concerning fermented foods, antifungal bacteria can be added as adjunct cultures during the process, along with the usual starters. This has been done in different foods including dairy products [87,88,89] and bakery products for which adjunct strains were added in the sourdough [90,91,92,93]. Alternatively, antifungal ingredients can be incorporated during the process or sprayed over the food, as done for bakery products [63]. In this study, only 12 out of 69 LAB and PAB strains selected from in vitro screening on whey flour agar showed an effect in situ after having sprayed the fermented medium on the surface of the bakery products, thus confirming the necessity to go beyond in vitro tests.

In most cases, fungal targets are inoculated and the ability of tested bioprotective agents to restrain fungal growth is evaluated. Their impact on mycotoxin production can also be investigated, as done for example for deoxynivalenol produced by *Fusarium culmorum* during malting [94] and aflatoxin produced in vitro by *Aspergillus flavus* and *Aspergillus parasiticus* [95]. Environmental challenge tests are also applied in which the activity is tested to prevent environmental fungal contaminants, after exposing the products to the airborne molds of the environment, for example in a bakery [83] or dairy environment [89]. The interest of appropriate in situ tests against a broad range of contaminants has recently been illustrated by studies using antifungal bacteria in bread bioprotection. Amongst the three *Lactobacillus* strains tested in vitro and in situ, the best performing strain in in situ environmental challenge tests was the least active in vitro [83]. In another study, the antifungal strain *L. amylovorus* DSM19280 was tested in comparison to a non-antifungal *L. amylovorus* strain to protect wheat and quinoa sourdough breads and clearly showed that the production of antifungal compounds varied according to the flour type [80].

### 3.2. Antifungal Microorganisms

A large number of microorganisms have been tested for their antifungal activity against food spoilers. This part mainly focuses on the microorganisms successfully tested as bioprotective tools in food products.

Lactic acid bacteria (LAB) are by far the main microorganisms tested for application in dairy and bakery productions, the two main food sectors studied for biopreservation against fungi. These microroganisms have also been tested in brewing (malting process), and in the manufacture of fermented vegetables or to protect grains, seeds, or fruits. For fruit bioprotection, the main candidates tested are bacteria, especially from the *Bacillus* group, and various yeast species. For fermented meats, yeast (*D. hansenii*) and *Penicillium* species have been tested against fungal spoilers. Many of these antifungal species have the characteristics of ‘microbial weeds’—i.e., species able to dominate communities that develop in open microbial habitats—a concept recently introduced for microorganisms by Cray et al. 2013 [96]. Table 1 lists the published literature on this subject over the past three years.

#### 3.2.1. Lactic Acid Bacteria

LAB encompasses a large and heterogeneous group of Gram-positive, low-GC, acid-tolerant bacteria, which produce lactic acid as the major metabolic end product of carbohydrate fermentation. LAB belong to the *Lactobacillales* order, which include 6 families, 36 genera, and more than 200 species. They are found in various biotopes such as environment, plants, human, and animal microbiota. They are largely used in the manufacture of a variety of fermented foods, where they contribute to improve shelf-life, organoleptic properties, and nutritional value. The main LAB in fermented foods correspond to species belonging to the *Lactobacillus*, *Lactococcu*s, *Leuconostoc*, *Carnobacterium*, *Enterococcus*, *Oenococcus, Pediococcus*, *Streptococcus, Tetragenococcus, Vagococcus*, and *Weissella* genera [28].

*Lactobacillus*, *Pediococcus*, and *Leuconostoc* species have been the most studied for their antifungal activity [24,25,140] and they have also been the most evaluated in situ in the past years (Table 1). Among them, *L. plantarum* is the most studied species representing about one third of reports on LAB and many different strains have been tested as antifungals in foods since 2013, (Table 1). *L. plantarum* strains and their metabolites have been tested in a wide range of foods where they inhibited different fungal species such as *Aspergillus*, *Penicillium*, *Rhizopus*, *Rhodotorula*, and *Pichia* spp. *L. plantarum* is a ubiquitous species [141] and is found in a wide range of ecological niches, such as milk [75], water for malt production[142], malted barley [143], or the aerial surfaces of plants or vegetables [33]. *L. plantarum* is also widely present in fermented foods, including different cheese varieties [144], and thus has the potential to be tested as an antifungal culture in a variety of applications, as already shown for the strain *L. plantarum* TK9 active against *Penicillium* in citrus, apple, and yogurt spoilage [117]. In another recent study*, L. plantarum* UFG 121 retarded the growth of *F. culmorum* in an oat-based beverage fermented with this strain [69]. There is a growing interest in looking for new antifungal strains in diverse types of fermented foods. With this in mind, *L. plantarum* strains with antifungal activities have been isolated from a variety of fermented foods including kimchi [98,112], koumiss[144], tempeh [145], and a number of other traditional fermented vegetables.[95,146].

Other species of lactobacilli have also recently been identified as potential antifungal cultures, such as *L. rossiae*[147], *L. amylovorus* [148], *L. harbinensis* [78], *L. brevis,* and *L. spicheri* [63]. Many other *Lactobacillus* species—including *L. rhamnosus*, *L. casei*, *L. paracasei*, *L. sanfranciscensis*, *L. fermentum*, *L. helveticus*, and *L. sakei*—added as adjunct cultures in fermented foods, have been shown to be able to extend the shelf life of various products: yogurt [87,88,89,142], fermented drinks [98] and bakery products [83,97].

*Leuconostoc* have also shown antifungal activities. *Leuconostoc* are used as starters in some fermented dairy products [149] but are also natural contaminants in various food products such as cheese and modified atmosphere packaged meat and seafood, where they can be responsible for spoilage [150]. In bakery products, *Leuconostoc* spp. exhibited good antifungal potential against fungal spoilers, with the highest proportion of active isolates against the 5 tested fungal targets, and 5 strains of *L. citreum* among the 10 most active LAB strains selected [63].

Concerning pediococci, several studies showed in vitro activity of *Pediococcus pentosaceus* strains against *Aspergillus flavus* and *Aspergillus niger* [151]. For example, 5 strains including 2 pediococci and 3 lactobacilli were selected from in vitro assessment of 13 strains based on their antifungal properties towards three ochratoxin A-producing *Aspergillus* species. One *Pediococcus* spp. strain was selected to be used as starter in cocoa fermentation inoculated with *A. carbonarius*, where it reduced fungal populations and toxin production [108].

Candidate antifungal bacteria to be tested in a given food product are often chosen among strains isolated from a similar biotope. However, successful results have also been obtained with strains tested in applications unrelated to their original biotope. For example, *L. amylovorus* DSM19280, a species commonly isolated from cereals or whole barley sourdough, inhibited the growth of *F. culmorum* that grows during the malting process and deoxynivalenol production, a mycotoxin produced by this common plant pathogen [152]. This LAB species also performed well in a bakery environmental challenge test [83]. Interestingly, the same *L. amylovorus* strain, added as an adjunct culture along with the usual lactic starters in Cheddar cheese manufacture, delayed *Penicillium expansum* growth by four days during ripening [89]. Similarly, a *L. reuteri* strain R29 isolated from human microbiota, was successfully used during the malting process to inhibit *F. culmorum* [94], and to extend the shelf life in rice and quinoa breads by incorporating *L. reuteri* in the sourdough [90]. These examples suggest that candidate strains selected for their antifungal properties from screening procedures could also be chosen without restriction of biotopes.

#### 3.2.2. Propionibacteria

Dairy propionibacteria (PAB) are also considered as antifungal candidates. The *Propionibacterium* genus, belonging to the *Actinobacteria* class, is a Gram-positive, high-GC content bacteria divided into ‘cutaneous’ and ‘classical’ (also referred to as ‘dairy’) propionibacteria based on their main isolation biotopes [153]. PAB and related species taxonomy has recently been reconsidered, with in particular ‘dairy’ PAB separated in the *Propionibacterium* genus consisting of *P. freudenreichii,* the main species used in cheese-making, and three other species, and a novel genus—*Acidipropionibacterium*—which encompasses the former species *P. acidipropionici, P. thoenii*, *P. jensenii*, and three other species [141].

In a large in vitro screening study on 197 dairy PAB strains, 13 strains—including 9 *P. jensenii, 2 P*. *acidipropionici*, and 2 *P. thoenii*—showed high antifungal activity against various yeast and mold species. In another study, in vitro screening revealed that almost all PAB strains were active in vitro against five mold species. Only two strains of *P. acidipropionici* and *P. freudenreichii* slightly delayed *A. niger* and *P. corylophilum* growth in milk bread rolls sprayed with antifungal cultures, whereas no inhibitory effect was observed in pound cake, probably because of the near neutral pH of this bakery product [63].

#### 3.2.3. *Bacillus* and Other Bacteria

Bacilli are Gram-positive, aerobic endospore-forming, and rod-shaped bacteria. They are found in diverse environments and are known to produce a variety of secondary metabolites including antimicrobial compounds. Their antimicrobial activities have been applied for the development of medical treatments but also more recently, as biocontrol agents of pre-harvest crop diseases and postharvest fruit and vegetable spoilage. In particular, *Bacillus subtilis* strains produce a wide variety of antimicrobial compounds, which include peptides and non-peptides [154].

Some recent bioprotection assays performed in situ using *Bacillus* species cells, endospores, CFS, or purified peptides on postharvest fruits illustrate the potential activities that they can exhibit (Table 1). *Bacillus* sp. strains present a large activity spectrum against pathogenic fruit molds from the *Aspergillus*, *Alternaria*, *Beltraniella*, *Botryosphaeria*, *Botrytis*, *Monilinia*, *Colletotrichum*, *Fusarium*, *Fusicoccum*, *Penicillium*, *Phomopsis*, *Phoma*, *Rhizoctonia*, and *Rhizopus* genera. Bioprotective activities were demonstrated in situ in a variety of fruits including tomatoes, apples, grapes, and pomegranates (Table 1). For example, *B. subtilis* AFB22 cells and CFS, selected after screening 200 antifungal *Bacillus* spp. isolates, prevented pomegranates from rotting when challenged with spores of *Phomopsis varsoniana* [119]. In another study, whole cultures of two *B.amyloliquefaciens* strains sprayed on Chinese jujube fruit inoculated with spores of three key pathogens of this fruit, *Phoma destructiva*, *Alternaria alternata* and *Fusicoccum* spp., reduced the disease incidence and induced different kinds of hyphal alterations on the fungal targets [120]. In vitro test using CFS from *B. amyloliquefaciens* also inhibited the growth of *B. cinerea*, a postharvest phytopathogen [155] and *Botryosphaeria dothidea* involved in peach gummosis [156].

Some *Bacillus cereus sensu lato* species have been recently proposed as bioprotective cultures and correspond to species commonly found in soil and foods but they may also be potential spoilers in dairy products and cereals and in some cases, harmful to humans. A *B. cereus sensu lato* strain isolated from an entomopathogenic nematode and its metabolites were active against an *Aspergillus* sp. in peanuts [118].

#### 3.2.4. Yeasts

If bacteria are well known for producing a large diversity of antimicrobial compounds, in recent decades, there has been a significant increase in interest regarding yeast antimicrobial properties. Yeasts are single cell microorganisms classified as members of the fungi kingdom. They are ubiquitous in the environment, are able to grow in a large variety of biotopes such as cereals, vegetables, fruits, meat, milk, as well as processed food products. Yeasts have been involved in food preservation for millennia, through the fermentation process of wine, beer, cereal-doughs, and certain cheese varieties, [157] where they contribute to organoleptic properties of fermented foods. Their antimicrobial activity is attributed to their ability to compete for nutrients, to acidify the medium, to resist stressful conditions (ethanol), but also to produce antimicrobial molecules named ‘mycocins’, also referred to as killer proteins affecting fungal growth [158]. Moreover, their capacity to colonize fruit, seeds, berries, leaves, and to compete for space and nutrients with other microorganisms, make them good candidates as biocontrol agents to limit postharvest decay for example [159]. Yeast antimicrobial activities and the numerous applications of mycocin-producing yeasts for preventing fungal spoilage in various foods and beverages such as wine, olives, beer, sake, miso, soy sauce, and salted vegetables have been reviewed by different authors (e.g. [160]). Nevertheless, these authors underlined that yeast strains must be used with caution since they could have a negative impact on the quality of end-products.

The yeasts *Meyerozyma guilliermondii*, *Candida fructus*, *Issatchenkia orientalis*, and *Candida quercitrusa* are frequently found associated with fruits or plant surfaces and showed antagonistic activities against fungal pathogens [161]. In a study on 11 yeast strains isolated from avocados, one *Wickerhamomyces anomalus* strain was able to inhibit *Colletotrichum gloeosporioides* and *C. acutatum* responsible for avocado anthracnose [128]. In the case of citrus fruit anthracnose, *Pichia membranifaciens*—alone or in combination with chitosan—inhibited *C. gloeosporioides* mycelium growth and spore germination [129]. *Hanseniaspora uvarum* isolated from grape surfaces has been reported as a natural preservative acting against the disintegration of gray post-harvest grapes caused by *B. cinerea* [127]. The same species was active against the green mold *P. digitatum* that spoils citrus fruits [85].

Antifungal yeast applications have also been suggested in the past three years for the production of dry-cured ham [136] and sausages [135] to control toxigenic penicillia populations that lead to spoilage inducing visual (black spots) and flavor defects, and above all that can produce ochratoxin A (OTA). In these studies, two *D. hansenii* strains revealed antifungal activity against *Penicillium verrucosum* and *P. nordicum* and were able to decrease OTA production. Paradoxically, *D. hansenii* can be involved in food spoilage as shown for some dairy products such as fresh cheese or cream but, at the same time, is food grade and used as a starter for specific dairy products. It is naturally found in meat products and on fruit surfaces and is used as a ripening culture to manufacture some surface ripened cheeses.

Foods are not the sole source of potential bioprotective yeasts, and yeasts with an antifungal potential have also been isolated from marine environments. This ecosystem confers unique properties to yeast matching with their potential use as bioprotective agents for postharvest fruits and vegetables. Antimicrobial activity screening of deep-sea fungi from the South China sea showed that 56% of fungal isolates exhibited antimicrobial activity against at least one pathogenic bacterium or fungus. Out of these antimicrobial fungi, the genera *Arthrinium*, *Aspergillus*, and *Penicillium* exhibited antibacterial and antifungal activities, while genera *Acremonium*, *Cladosporium*, *Geomyces*, and *Phaeosphaeriopsis* displayed only antifungal activity [162]. The marine yeast, *Rhodosporidium paludigenum*, isolated from the East China Sea, effectively inhibited *P. expansum* on pear fruit, and *Alternaria alternata* on jujube fruits [163]. A number of bioactive metabolites produced by deep-sea fungi have been recently reviewed, thus more than 180 bioactive secondary metabolites derived from deep-sea fungi have been documented in the literature, including compounds with antifungal activity [66].

#### 3.2.5. Filamentous Fungi

As previously stated, not all filamentous fungi are responsible for food spoilage and postharvest crop disease. Some of them contribute to the flavor and typical visual characteristics of some fermented foods—such as surface-ripened cheese [164], fermented sausage [50], and ham [138]—and can also be used as antifungal agents, in particular to protect the surface of dry-ripened products where the low a_w_ prevent the use of antifungal LAB. Some filamentous fungi such as the *Penicillium* species *P. nalgiovense* (non-toxigenic) and *P. chrysogenum* are both commercial cultures commonly used in the meat industry [165]. Both species have recently been tested against *Aspergillus* and *Penicillium* mycotoxin producers*. P. chrysogenum* strains isolated from dry-cured ham inhibited two common spoilers *A. flavus* and *P. restrictum* in vitro [166,167] by the production of an antifungal protein, whereas *P. nalgoviense,* from the commercial culture TEXEL PN1, limits the growth and OTA production of *P. verrucosum* [137]. In another study of the bioprotective role of *P. chrysogenum,* it was suggested to combine the use of *P. chrysogenum* CECT 20922 as bioprotective culture and the reduction of a_w_ throughout the ripening process to avoid black spot formation in dry-cured ham [138].

As will be detailed below, fungi produce antimicrobial peptides (AMP) that are a valuable strategy to avoid fungal spoilage and mycotoxin production.

## 4. Action Mechanisms of Antifungal Microorganisms

**Figure 1:**
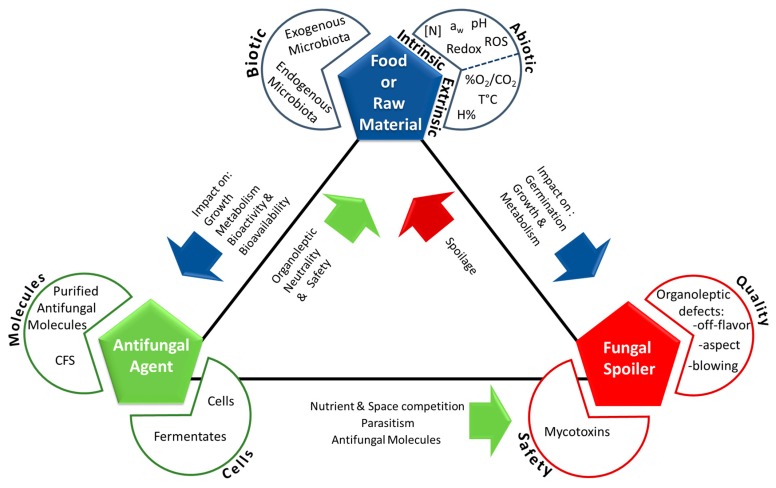
Diagram representing possible interactions occurring between food or raw material, antifungal agents, and fungal spoilers in a biopreservation context (CFS: culture-free supernatant, ROS: Reactive Oxygen Species, [N]: nutrient content, H%: hygrometry).

Fungal spoilage control using antifungal microorganisms is a complex task which success depends on network of the interactions between three main actors: the food itself (comprising its natural microbiota), the fungal spoiler(s), and the antifungal microorganism(s) (Figure 1). Depending on food type and antifungal microorganism, different action mechanisms—combined or not—can be responsible for spoilage fungi inhibition.

Antifungal molecules can have (i) a target-specific mode of action, such as natamycin which blocks fungal growth via a specific interaction with ergosterol in the membrane, or (ii) a non-specific mode of action. In the latter case, molecules can generate an acid (pH) and/or an osmotic stress, which can draw water from the cytoplasm, or correspond to chaotropic and hydrophobic stressors that can weaken or inhibit non-covalent interactions between macromolecular systems by reducing water [168]. Chaotropicity, like water activity and pH, could be a key parameter in preservation.

### 4.1. Action Mechanisms of Antifungal Lactic Acid Bacteria and Propionibacteria in Fermented Foods

Antibiosis (e.g., production of antifungal molecules) and pH decrease are the main factors contributing to LAB and PAB antifungal activity in foods. Indeed, LAB and PAB produce lactic and propionic acids as main fermentation end-products, respectively. Moreover, most antifungal LAB, being facultative or strictly heterofermentative lactobacilli and pediococci, are also able to produce acetate from pentose (facultative or strictly heterofermentative LAB) and ethanol, or acetate (in the absence or presence of O_2_ or other electron acceptors, respectively) and CO_2_ from hexose (strictly heterofermentative LAB). On the other hand, propionibacteria also produce acetic and succinic acids as well as CO_2_ through the transcarboxylase cycle, in molar ratios depending on the substrate, environmental conditions, and strain [169]. These organic acids, synthesized at important levels in food (e.g., in g/L or g/kg order), possess antifungal activities, especially acetate and propionate, whose minimum inhibitory concentrations (MICs) are 30 to 100 times inferior to that of lactate depending on the fungal target. Moreover, acetate MIC is reduced in the presence of lactate, indicating that they can act in synergy. Nevertheless, the production of the aforementioned acids is not sufficient to explain antifungal activity of LAB and PAB [170].

For antifungal LAB, many molecules, produced at low quantities (mg/L or mg/kg) and below their individual MIC, are also likely to act in synergy with lactic and acetic acids. The nature and quantity of these compounds is species- and strain-dependent and so far, a very large array of molecules has been described in the literature [24]. They include other organic acids (hydrocinnamic acid, dl-β-phenyllactic acid, dl-β-hydroxyphenyllactic acid, polyporic acid, azelaic acid, 2-hydroxybenzoic acid*,* 4-hydroxybenzoic acid, *p*-coumaric acid, vanillic acid, caffeic acid, succinic acid, 2-pyrrolidone-5-carboxylic acid), fatty acids (decanoic acid, 3-hydroxydecanoic acid, (*S*)-(−)-2–hydroxyisocapric acid, coriolic acid, ricinoleic acid), cyclopeptides [cyclo(L-Pro-L-Pro), cyclo(L-Leu-L-Pro), cyclo(L-Tyr-L-Pro), cyclo(L-Met-L-Pro), cyclo(Phe-Pro), cyclo(Phe-OH-Pro), cyclo(L-Phe-L-Pro), cyclo(L-Phe-trans-4- OH-L-Pro), cyclo(L-His-L-Pro), and cyclo(Leu-Leu)], reuterin, hydrogen peroxide, and volatile compounds such as diacetyl [101,171,172,173,174,175,176,177,178]. For example, Miezkin et al. (2017) [175] attributed the antifungal effect of *L. harbinensis* K.V9.3.1.Np to the synergistic action of acetic, lactic, 2-pyrrolidone-5-carboxylic, (*S*)-(−)-2–hydroxyisocapric, and 2-hydroxybenzoic acids, while Aunsbjerg et al. (2015) [101] showed that increased production of diacetyl and to a smaller extent, 2,3-pentadione, acetic acid, and butanoic acid were involved in the antifungal activity of *L. paracasei* DGCC 2132*.* In another study, Le Lay et al. (2016) [179] showed that lactic, acetic, and propionic acids, ethanol and hydrogen peroxide, as well as other compounds present at low levels such as dl-β-phenyllactic, dl-β-hydroxyphenyllactic, azelaic, and (*S*)-(−)-2–hydroxyisocapric acids were responsible for the antifungal activity of lactobacilli and propionibacteria CFS active against spoilage molds in bakery products. A fraction extracted from a culture of *Lactococcus* sp. BSN307, that contained 2,4-di-tert-butylphenol, was effective for wheat grains preservation from the attack by *A. niger* and different *Fusarium* sp. [110]

For propionibacteria, the four dairy species (*P. freudenreichii*, *P. acidipropionici*, *P. thoenii*, and *P. jensenii*) also produce dl-β-phenyllactic acid .[77] Propionicin PLG-1, a 9 328 Da bacteriocin produced by *P. thoenii* P127, is active against various bacteria but also yeast and mold species [180]. Several antimicrobial organic acids acting in synergy have been identified in a protective mixed culture of *P. jensenii* SM11 and *L. paracasei* strain including 2-pyrrolidone-5-carboxylic, dl-β-phenyllactic, and dl-β-hydroxyphenyllactic acids [181]

The action mode of organic acids is quite well understood [182]. Organic acids, under their undissociated form, can diffuse through the microorganism membrane and dissociates in the cell, thereby causing a decrease in intracellular pH. The accumulation of toxic ions combined with membrane disruption, inhibition of essential metabolic reactions, and/or stress in intracellular pH homeostasis may finally lead to cell death. In this so-called ‘weak acid theory’, the acid pKa value and the pH of the medium are therefore important factors influencing antifungal activity of organic acids. Indeed, the higher the pKa and the lower the medium pH, the more the acid will be in its undissociated form and the higher will be its effect on intracellular pH and antifungal activity. However, not all organic acids act this way. For example, sorbic acid was shown to inhibit plasma-membrane H^+^-ATPase proton pump but not to decrease intracellular pH in *S. cerevisiae* [183]. It is also worth mentioning that phenolic compounds (4-hydroxy benzoic acid, vanillic acid); acetic, propanoic, and butanoic acids; acetoin; ethanol; and other volatile compounds can act as chaotrophic stressors [168,184,185].

It should be highlighted that the multiplicity and variety of antifungal molecules identified so far in LAB or PAB render difficult their exhaustive identification and quantification. Therefore, in future studies, it would be desirable to conduct large targeted- and untargeted assays for antifungal molecule identification and quantification, by combining different extraction and analysis methods, such as LC-MS/MS or GC-MS. In this context, Brosnan et al. (2012, 2014) [186,187,188] developed two methods to extract and simultaneously detect and quantify a large variety of antifungal compounds. The method was further slightly modified and applied by Le Lay et al. (2016) [179]. Future work should also include the investigation of individual and combined effects of mixtures of antifungal molecules identified so far. Chaotrophicity could also be taken into account to determine the mechanism of action of antifungal microorganisms, nevertheless further studies must be done to determine the chaotrophicity of the mixture of identified molecules produced at low concentration by antifungal microorganisms of interest in food. Concerning their action mode against different fungal targets, only few studies looked at the effects of antifungal molecules produced by bioprotective LAB on fungal physiology in the food context. Crowley et al., (2013)[189] investigated the response of *Aspergillus fumigatus* Af293 (at the transcriptomic and morphological levels) to antifungal molecules present in *L. plantarum* 16 CFS. These authors observed an altered transcription of a large variety of cellular functions involved in metabolism, transport, signaling, ergosterol biosynthesis, and cell stress, including that of LaeA, a global regulator of secondary metabolism. More recently, Mieszkin et al. (2017) [175] investigated the action mechanisms involved in the bioprotective effect of *L. harbinensis* K.V9.3.1.Np against *Y. lipolytica* in fermented milk. The CFS obtained after milk fermentation by yogurt starters in co-culture with the bioprotective strain had a fungistatic rather than a fungicidal effect accompanied with a significant loss of cultivability in the studied yeast. It also led to membrane depolarization and intracellular pH decrease, as well as morphological changes including membrane collapsing and cell lysis.

### 4.2. Action Mechanisms of Antifungal Yeasts and Molds in Fermented Foods

As mentioned in the previous sections, selected yeasts and molds can be used as antifungal agents in dry-fermented foods to inhibit the growth of undesirable fungi. Their abilities to compete for space and nutrients with spoilage fungi as well as to produce antifungal molecules such as antifungal proteins and volatile compounds explain their antifungal properties [26]. Núñez et al. (2015) [136] showed that *D. hansenii* isolates are active against spoilage molds from dry-cured ham produced 2-methyl-1-butanol and other volatiles as well as unidentified diffusible molecules which were active against *P. verrucosum*. *M. guilliermondi* LCF1353, in combination with added *L. plantarum* 1A7 and *W. anomalus* 1695 strains that were previously selected for their antifungal activity against *P. roqueforti* in wheat flour bread [92] produced ethyl acetate and a β-1,3-glucanase during dough fermentation, which were efficient in preventing fungal contamination of bread slices without negatively affecting bread sensorial properties [190]. As recently reviewed by Delgado et al. (2016a, 2016b) [26,191] molds can produce various peptides and proteins with antifungal properties, including basic, cysteine-rich antifungal proteins (AFPs) with a 5.5–10 kDa molecular weight. Their structure makes them highly stable to low pH, heat, and proteolysis which is compatible with their possible antifungal role in fermented foods such as cheeses and fermented meats. After binding the cell wall or being internalized by target fungi, AFPs can alter chitin synthesis leading to cell death, or increase intracellular reactive oxygen species (ROS) levels, leading to cell permeabilization and apoptosis [26].

### 4.3. Action Mechanisms of Antifungal Yeasts and Bacillus spp. for Control of Postharvest Diseases

Biopreservation agents used for controlling postharvest disease have complex action mechanisms including competition for nutrients (e.g., iron and carbon sources) and space [71,130,192], antibiosis (e.g., production of antifungal molecules such as peptides and lipopeptides and volatile organic compounds) [24,101,193,194,195], mycoparasitism associated with lytic enzyme production (e.g., glucanases, chitinases, and proteases) [157,195] and induced host resistance [29].

For example, the group of cyclic lipopeptides (surfactins, fengycins, and iturins) produced by *B. subtilis* and other *Bacillus* sp. strains have been shown to protect host plants from a number of pathogens including fungi [154,193]. Cyclic lipopeptides possess surface properties that are believed to contribute to the ability of *B. subtilis* cells to spread and colonize surfaces, whereas the fengycin and iturin molecular families are strongly toxic for fungi [154,196]. New antifungal peptides are continuously characterized, as for example, a large peptide synthetized by *Bacillus subtilis* B25, which inhibits the growth of *Fusarium oxysporum* f. sp*. cubense*, a pathogen infecting bananas [197].

Volatile antifungal compounds that can act at a distance are produced by antifungal species, thus opening new possibilities to control food spoilage such as biofumigation, provided that the safety of this mode of application is ensured [198]. A variety of antifungal volatiles have been identified—including 2-phenylethanol, 2-methyl-1-butanol, 3-methyl-1-butanol, 2-methyl-1-propanol, 5-pentyl-2-furaldehyde, 2-nonanone, and 2-ethyl-1-hexanol [18], 2-Phenylethanol was identified for example as one of the antifungal volatiles produced by a strain of *P. expansum* R82 active against different species of postharvest fungal pathogens [199] and its mechanisms of action against *P. digitatum* and *P. italicum* investigated [200].

Many different mechanisms of action were identified in yeasts active against grape pathogens. The most important are the production of laminarinases, antifungal volatiles or growth-inhibiting metabolites, the inhibition of fungal spore germination, decrease of germinal tube length, and the competition for carbon sources and iron [201]. At least two of the mechanisms of action were present in the yeast isolates assayed, with many different ‘antifungal patterns’ found according to the isolates. In *Pichia anomala* strain WRL-076, used as a biocontrol agent to reduce aflatoxin contamination of tree nuts, 2-phenylethanol was identified as an antifungal volatile compound, which inhibited spore germination, growth, and aflatoxin production in *A. flavus* [202]

These aspects were recently reviewed in detail by Spadaro and Droby (2016) [18]. Di Francesco et al. (2016) [18] as well as Rahman (2017) [203]. Biofilm formation, quorum sensing, and oxidative stress were also underlined in these reviews as other important key factors in the action mode of these biocontrol agents. These reviews also pointed out the potential of omics techniques to study antagonist-pathogen-host interactions.

## 5. Implementation of Biopreservation Methods against Fungal Spoilage

### 5.1. Optimization and Application Modes

Once antifungal cultures have been selected and/or the molecules supporting the activity have been identified, the next questions that can be raised concern (i) the potential enhancement of their activity in terms of efficiency or action spectrum (i.e., the number of inhibited species/strains) and (ii) the antifungal product type and its application mode.

Concerning optimization of the antifungal activity, several methods have been developed to enhance the production of bioactive molecules or extend their action spectrum, and correspond to the use of microorganisms in co-cultures, addition of enhancing molecules as precursors to trigger their biosynthetic pathways, induction of stress conditions to cultures, or to associate microbial cultures with other active molecules. For example, mixed fungal cultures using *Phomopsis* sp. K38 and *Alternaria* sp. E33 [204] were shown to create a new cyclic tetrapeptide (cyclo-[l-leucyl-*trans*-4-hydroxy-l-prolyl-d-leucyl-*trans*-4-hydroxy-l-proline]), which was shown to be active against *Gaeumannomyces graminis*, *Rhizoctonia cerealis*, *Helminthosporium sativum*, and *Fusarium graminearum* in vitro. Co-cultures can also positively stimulate antifungal activity as observed with a kefir symbiotic consortium combining three bioprotective LAB species (*L. plantarum*, *L. kefir*, and *Lactococcus lactis* subsp. *lactis)* that was able to extend shelf life of a maize food product called ‘arepa’ against *A. flavus* contamination [113]. The use of an association of antifungal strains each exhibiting antifungal activity towards different fungal targets may be a way to enlarge the antifungal spectrum of action. Another means to enhance bioprotective activities is the addition of certain substrates in the medium or food product. Certain substrates can potentially inhibit antifungal microorganism development while others may enhance their activity. Glycerol is known to enhance the antifungal activity of LAB. For example, the use of immobilized cells of *L. reuteri* in a glycerol based medium enhanced reuterin production [102], a widely used preservative in foods. In the same way, Le Lay et al. [63] observed that the antifungal activity of *L. brevis* Lu35 and *L. reuteri* 5529 was increased on wheat based medium supplemented with 2.5% olive oil and 150 mM glycerol, respectively. Other examples correspond to the use of sorbitol [205] or xylitol or galactosyl-xylitol [115] with *Lactobacillus* strains to inhibit a wide spectrum of fungi in vitro, while no inhibition was observed if other media were used. Then, stress conditions such as temperature, pH, nutrient availability, and cell population density during culture of antifungal microorganism can trigger metabolic pathways that enable the bacterium to cope with the stressful environment [206] which leads to the production of diverse metabolites including antifungal molecules.

Finally, the association of bioprotective cultures with other molecules, such as chitosan, has been described and is currently used to protect postharvest crops by the coating technique. Chitosan protects fruits and vegetables from fungal decay, creates a barrier with the environment and delays ripening. A study by [129] tested the coating of citrus fruits with a chitosan mixture containing a *P. membranifaciens* culture to prevent *C. gloeosporioides* fungal infections which was more effective than using the yeast culture alone. Similarly, 2-hydroxybenzoic acid enhanced the biocontrol efficacy of the yeast *R. glutinis* against *P. expansum* and *A. alternata* in cherry fruit [207] Finally, the role of other ingredients in foods to help extend product shelf-life is also important. The use of spices combined with an antifungal LAB culture to efficiently prevent fungal spoilage of an Ethiopian spiced fermented cottage cheese was recently described [208].

Concerning the antifungal agent type, they correspond to the use of cells (as adjunct cultures), their fermentates, CFS, or partially or totally purified compounds, the best example being natamycin, a universal antifungal agent from *Streptomyces natalensis*. The type of antifungal agent combined with the targeted food matrix will then determine the application mode. Legislative aspects may also have to be taken into account. Application modes mainly correspond to the addition in the product as adjunct culture in fermented products or ingredient, spraying, dipping, or drenching. In bakery products, Le Lay et al. (2016) [63] used two different application modes for challenge tests. In milk bread rolls, a fermented product, cell cultures were added to the classical starters and showed that, for some strains, the antifungal activity was conserved despite the baking process. For pound cake, a non-fermented product, bioprotective action was performed through surface spraying. Also, *L. amylovorus* DSM19280 used as a starter in the preparation of gluten-free quinoa bread proved to extend the mold free shelf-life [80] The protection of post-harvest crops and cereals can be performed by dipping food in the active culture or cell suspension as shown by Pantelides et al. (2015) [70] with wounded grape berries and Perez et al. (2016) [131] with lemons, for biocontrol of *B. cinerea* and *P. italicum*, respectively. As for spraying, pulverization of lactic acid bacteria strain cell suspensions over wounded and non-wounded apples protected the fruits from *P. expansum* colonization [116].

### 5.2. Constraints

As previously stated, due to ever increasing societal demands for preservative-free foods, the use of antifungal cultures for biopreservation has triggered a strong interest in the scientific community. However, although numerous studies have been reported in the literature, only few commercial solutions are available to date (Table 2). This is due to several constraints that may impair the commercialization of candidate antifungal cultures.

The first constraint concerns the gap between an observed activity in a culture medium (the in vitro effect) and the actual activity in the final matrix (in situ effect). Several studies have described that numerous microorganisms can show interesting results in in vitro screening conditions but that the actual number drastically decreases when tested in the target food matrices. For example, Delavenne et al. (2013) [78] tested 11 bacteria exhibiting important antifungal activity in vitro and only 1 strain. belonging to the *L. harbinensis* species, proved to be active in yogurt. One way to get around this problem is to use semi-synthetic media that can closely mimic food composition. However, as shown by Le Lay et al. (2016) [63] the use of a wheat based medium, although more effective than MRS for antifungal activity expression, did not completely represent the complexity of bakery products and therefore, the number of efficient antifungal strains was still far smaller when tested on sweet bread and pound cake. The observed differences are obviously linked to the complexity of the food or raw material matrices. They are characterized by different abiotic and biotic factors that can impact the growth and metabolism of the antifungal cultures or the bioavailability and bioactivity of antifungal compounds. The fungal spoiler load, the antifungal agent load or concentration, and the treatment moment will also impact efficiency. The interactions between fungal spoilers, food matrices, and antifungal agents are illustrated in Figure 1. Therefore, it is crucial to test the actual antifungal efficiency of potential bioprotective strains on the final product intended for use (challenge-tests).

Direct testing on the food matrix also enables the study of another important constraint linked to the direct addition of bacteria into a given food matrix. Indeed, this method directly provides information related to its impact on the organoleptic qualities of the product. According to its metabolism, an antifungal culture may exert a positive, neutral, or negative impact. Some examples are as follows, heterofermentative antifungal microorganisms may lead to CO_2_ production directly affecting the product’s aspect (bubble or air-pocket formation) or the packaging (blown packages), while other microbial candidates may exhibit intense enzymatic activities (i.e., proteolytic, amylolytic, or lipolytic) directly affecting product texture and overall aspect. Finally, other antifungal strains may produce aroma compounds leading to off-flavor defects. In this context, antifungal culture selection should aim towards organoleptic neutrality. This can be achieved by the use of sensory evaluation methods (i.e., triangle test, rapid methods for sensory profiling) to define and quantify a potential organoleptic impact in the final product. Notably, the quantity of antifungal agent to be added for efficient activity may be incompatible from an organoleptic but also a cost point of view.

The safety of the selected cultures or their CFS is another key aspect beyond these first two constraints linked to antifungal culture efficiencies and product quality issues. Safety assessment, including any regulatory considerations that need to be addressed, has now become a key step during strain selection for biotechnological use in general. In this sense, the European Union developed the Qualified Presumption of Safety (QPS) approach as a means to assess the safety of a broad range of biological agents with intended uses as sources of food and feed additives, enzymes, and plant protection products [209] and can more generally be considered as a premarket evaluation of microorganisms used in food and feed production [210] to ensure their safe use. In the United States, a similar ‘Generally Recognized as Safe’ (GRAS) status exists for food and substances used in food [211]. This status implies that a given GRAS substance has been efficiently shown to be safe under the conditions intended for its use. In both cases, regularly updated lists of QPS recommended biological agents or GRAS food substances are provided by the corresponding regulatory bodies (EFSA and FDA, respectively). In the case of bioprotective cultures, fermentates, or CFS, the goal is to intentionally and safely add them to a food matrix for their bioprotective properties while ensuring consumer safety. In this context, it is necessary to follow a rigorous safety assessment procedure, taking multiple criteria into account and a model has recently been proposed for LAB and propionibacteria safety evaluation by Coton et al. [212]. First of all, according to the strain intended for direct use or the strain used to produce the fermentates/CFS, the following criteria may be considered (i) overall body of knowledge including history and intended end use; (ii) well-defined taxonomy with identification at the species level; and (iii) any potential safety hazards including pathogenicity factors, unwanted antibiotic resistance profiles (especially acquired resistances via horizontal gene transfer events that can potentially lead to further dissemination of this trait), and undesirable compound formation including biogenic amines, allergens, or toxins [209,210,211,212,213,214,215]. In the case of the most commonly considered bacterial groups for industrial applications (i.e., LAB and propionibacteria), many members of these groups already have a long history of safe use in foods and/or may already have a GRAS or QPS status. For such cultures, the safety criteria that need to be included during evaluation can be reduced to the current body of knowledge and intended end use, well-defined taxonomy (although in some cases, this may include very recently described species), antibiotic resistance profiles, and biogenic amine production (no known toxins or virulence factors have been described so far). Biogenic amines produced by LAB species in fermented foods, including dairy products, have been well reviewed in the literature (see [216,217,218]). Their production has been described as a strain-dependent trait for many species [219,220] which emphasizes the need to include this feature during safety assessment. By following this safety assessment procedure, a given culture or corresponding fermentate/CFS intended to be used as a bioprotective agent may be excluded for safe use if multi-resistant antibiotic profiles are identified (in particular, any acquired resistances and/or the presence of the corresponding biosynthesis genes and mobile elements in the genome) or if any undesirable compounds such as biogenic amines (especially tyramine, histamine, putrescine, or cadaverine) potentially known to have a negative physiological effect on sensitive consumers [218,221,222] and/or if the corresponding biosynthesis genes are identified. In the case of other bacterial groups (i.e., *Bacillus*) or fungal strains (yeasts or molds), safety assessment should also include determining whether the strain produces other undesirable compounds (especially toxins by bacteria or mycotoxins by molds). If detected, this would exclude their use in industrial applications.

Other constraints/criteria that must be considered before marketing a candidate bioprotective culture—or its corresponding fermentate/CFS—are related to strain selection, propagation, and preparation (i.e., selling format). As mentioned above, only few commercially available bioprotective cultures are on the market and this is likely due to the fact that not all criteria have been thoroughly studied or satisfied. Indeed, multiple requirements must be met, on top of the constraints mentioned above, knowing that any given bioprotective agent will be intentionally added to the food matrix at different steps during food processing and bioprotective activities must be conserved during shelf life. Preliminary tests to evaluate propagation to high population, microbial stability, and cell viability after preparation (in the selling format) and addition into the food matrix are required and bioprotective activity must also be maintained during the storage period [29,87,223,224]. This is an essential step as stability and/or activity may be altered according to how the protective culture is prepared. Different techniques can be used to prepare dry or liquid formulations, such as freeze-drying or spray drying techniques to produce active dry powder and metabolite(s) purification procedures. Dry formulations have been shown to be more advantageous for many reasons including longer shelf life, easier storage under non-refrigerated conditions, and ease of distribution [223,225]. In all cases, the impact of preparation procedures on the efficiency of protective cultures/fermentates/CFS should be determined. The most important criteria is to ensure that adequate shelf life is retained to ensure efficient use as a bioprotective agent in the food matrix. Notably, previous studies have shown that environmental conditions encountered in foods can have either positive or negative effects on bacterial antimicrobial activities [226,227,228,229,230] which again emphasizes the need for thorough in situ evaluation of candidate protective cultures before commercial use.

Finally, another constraint can be at the regulation level as, in some countries, the regulation definition of these antifungal agents (starter, ingredient, technological auxiliary, or even additive) can be raised. However, as discussed previously, the main antifungal agents correspond to microorganisms (or product of their metabolism) with a long history of use and GRAS or QPS status, thus guarantying an expected safety of use, provided that all mentioned safety aspects have been covered.

## 6. Conclusions

The increasing societal demand for less processed and more natural food products—while conserving those products’ quality, safety, and shelf-life—has raised the question of chemical preservative replacement. In this context, bacteria and fungi as well as their metabolites are natural alternatives of interest for use in food as bioprotective tools to fight fungal spoilage and to answer consumer demands and legislation. From an applied point of view, the difference between the number of studies and the number of available microbial cultures indicates that efforts are needed to facilitate their application in food commodities. One of the main aspects concerns the crucial role of in situ studies using adapted fungal targets during antifungal activity screening or confirmation processes. Also, safety assessment, organoleptic neutrality, and activity stability of the bioprotective cultures need to be evaluated prior to marketing. From a cognitive point of view, while antifungal compounds have been widely studied, and have generally been shown to act synergistically, there is still a lack of knowledge concerning the overall picture as to what molecules are involved and their action mechanism(s). The combination of pertinent biochemical analytic tools and omics methods should enable us to decipher antifungal action mechanisms, potentially identifying new levers for antifungal activity. Finally, if finding natural antifungal agents is a key factor, it can only be considered in good practices and within the HACCP context as one of the hurdle technologies to prevent fungal spoilage.

## Figures and Tables

**Table 1:** Active bioprotective cultures against fungal contaminants of postharvest fruits and processed and raw foods.

Food Field	Group	Antifungal Microorganisms (Active In Situ/Tested Strains)	In Situ Test	Source of Microorganism	Application Method	Activity Spectrum (Inhibited/Tested)	Reference
bakery	LAB	*Lactobacillus brevis* ITM18	yeast-leavened bread	sourdough	CFS as ingredient	*Aspergillus niger*	[97]
bakery	LAB	*Lactobacillus plantarum* HD1	Korean draft rice wine	kimchi	CFS	*Aspergillus fumigatus* and *Pichia kudriavzevii*	[98]
bakery	LAB	*Lactobacillus amylovoru*s DSM19280 (1/1)	sourdough quinoa bread	cereal isolate	cells in sourdough	environmental molds	[80]
bakery	LAB	*Lactobacillus plantarum* CRL778	wheat bread	homemade wheat dough	SL778: fermentate as ingredient	environmental molds	[99]
bakery	LAB	*Lactobacillus plantarum* UFG 121 (only 1 in situ from best 2/88 in vitro)	oat-based product	food	cells in sourdough	*Fusarium culmorum* (only 1 tested in situ), *Penicillium chrysogenum, Penicillium expansum, Penicillium roqueforti*, and *Aspergillus flavus* (5/7 in vitro)	[69]
bakery	LAB	*Lactobacillus amylovorus* DSM19280 (1/3)	sourdough wheat bread	cereal isolate	cells as starter	*Fusarium culmorum*	[83]
bakery	LAB	*Lactobacillus reuteri* R29, *Lactobacillus brevis* R2, *Lactobacillus amylovorus* DSM19280	sourdough of quinoa and rice bread	human, pork, and cereal	cells in sourdough	environmental molds	[90]
bakery	LAB	*Lactobacillus bulgaricu*s CECT 4005, *Lactobacillus plantarum* CECT 749 (active in situ 2/6), *Lactobacillus johnsonii* CECT 289, *Lactobacillus rhamnosu*s CECT 288, *Lactobacillis ruminis* CECT 1324, and *Bifidobacterium bifidum* CECT 870T (6 active in vitro/16)	loaf bread	not detailed	cells in sourdough	*Aspergillus parasiticus* (only one tested in situ) and *Penicillium expansum*	[100]
bakery	LAB & PAB	*Leuconostoc citreum* (5 strains)*, Lactobacillus sakei, Lactobacillus plantarum, Lactobacillus spicheri* O15*, Lactobacillus reuteri* 5529*, Lactobacillus brevis* Lu35*, Propionibacterium acidipropionici* and *Propionibacterium freudenreichii* LSaci68 (by surface-spraying 12 LAB/69)	pound cake and milk bread roll	milk, milk roll sourdough, and others not detailed	whole culture as sourdough ingredient for milk bread roll and sprayed	*Cladosporium sphaerospermum* and *Wallemia sebi* on pound cake; and *Eurotium repens*, *Aspergillus niger*, and *Penicillium corylophilum* on milk bread roll	[63]
dairy	LAB	*Lactobacillus harbinensis* K.V9.3.1Np, *Lactobacillus. rhamnosus* K.C8.3.1I, and *Lactobacillus paracasei* K.C8.3.1Hc1 (3/11)	yogurt	cow and goat milk	cells as adjunct culture	*Debaryomyces hansenii*, *Kluyveromyces lactis*, *Kluyveromyces marxianus, Penicillium brevicompactum*, *Rhodotorula mucilaginosa*, and *Yarrowia lipolytica* (6/6)	[78]
dairy	LAB	*Lactobacillus casei* AST18 (1/1)	yogurt	Chinese dairy products	cells as adjunct culture	*Penicillium* sp. (1/1)	[88]
dairy	LAB	*Lactobacillus paracasei* DCS302	yogurt	not detailed	cells as adjunct culture	*Penicillium* sp. nov. DCS 1541, *Penicillium solitum* (2/8)	[101]
dairy	LAB	*Lactobacillus harbinensi*s K.V9.3.1Np (1/2)	yogurt	cow milk	cells as adjunct culture	*Yarrowia lipolytica* (1/1)	[87]
dairy	LAB	*Lactobacillus reuteri* INIA P57	semi-hard ewe milk cheese	pig feces (isolated by Langa 2003)	cells as adjunct culture supplemented with glycerol	Not evaluated	[102]
dairy	LAB	*Lactobacillus amylovoru*s DSM 19280 (1/1)	cheddar cheese	cereal environment	cells as adjunct culture	*Penicillium expansum (*1/1) and environmental molds	[89]
dairy	LAB	12 strains of *Lactobacillus plantarum* (12/897)	cottage cheese	fresh herbs, fruits, and vegetables	cells as added to the finished product	*Penicillium commune*	[103]
dairy	LAB	*L. rhamnosus* A238*, L. rhamnosus* A119 (2/5) *The association of L. rhamnosus* A238 with *B. animalis* subsp*. lactis* A026, and *L. rhamnosus* A119 with *B. animalis* subsp. *lactis* A026	cottage cheese	not detailed	cells added to the finished product	*Penicillium chrysogenum* (1/1)	[104]
malting	LAB	*Lactobacillus brevis* R2Δ (1/1)	barley malt extract fermentation	porcine isolate	cells as starter	Not evaluated	[105]
malting	LAB	*Lactobacillus brevis* R2Δ and *Lactobacillus plantarum* FST1.7 (2/2)	barley malt extract (wort) fermentation	porcine and barley isolate	cells as starter	*Fusarium culmorum*	[106]
malting	LAB	*Lactobacillus brevis* R2Δ (1/1)	barley in malting process	porcine isolate	cells as starters and CFS	*Fusarium culmorum* and *Fusarium graminearum*	[107]
malting	LAB	*Lactobacillus reuteri* R29 and *Lactobacillus amylovorus* DSM19280	malting process (steeping and germination)	human and cereal isolates	CFS (wort as growth media) as the steeping liquor	*Fusarium culmorum*	[94]
fermented vegetables	LAB	*Pediococcus* spp. A19 (tested in situ) *Pediococcus* spp. A21, *Lactobacillus plantarum* B4496, *Lactobacillus brevis* 207, and *Lactobacillus sanfranciscensis* BB12 (5/13)	cocoa	fermenting cocoa	cells as starter	*Aspergillus carbonarius*, *Aspergillus niger*, and *Aspergillus ochraceu*s	[108]
fermented vegetables	LAB	*Lactobacillus fermentum* YML014	tomato puree	*gari*, fermented cassava (starchy root)	cells	*Penicillium expansum* (only one tested in situ), *Aspergillus flavus*, *Aspergillus niger*, *Candida albicans*, and *Zygosaccharomyces rouxii* (low inhibition of yeasts)	[109]
fermented vegetables	LAB	*Lactobacillus helveticus* KLDS 1.8701 (1/4 also *L. helveticus*)	fermented soybean milk	dairy products	cells as adjunct culture	*Penicillium* sp. (1/1)	[81]
grain/seed	LAB	*Lactococcus* sp. BSN307	wheat grains	rotten jackfruit, guava, and animals fecal samples	submerged in purified volatile organic compound 2,4-di-tert-butylphenol	*A. niger*, *F. moniliforme*, *F. graminearum*, *F. chlamydosporum,* and *F. oxysporum*	[110]
grain/seed	LAB	*Lactobacillus plantarum* LR/14	wheat seeds (*Triticum aestivum* var. HD 2824)	not detailed	AMP LR14 solution	*Aspergillus niger*, *Rhizopus stolonifer*, *Mucor racemosus*, and *Penicillium chrysogenum.*	[111]
grain/seed	LAB	*Lactobacillus plantarum* YML007 (1/1400)	soybean	kimchi	CFS	*Aspergillus niger*	[112]
grain/seed	LAB & Fungi	kefir grains contain a symbiotic consortium of LAB and yeasts (*Lactobacillus plantarum*, *L. kefir*, *Lactococcus lactis* subsp. *lactis*, *Saccharomyces* and *Acetobacter*)	arepa (corn cakes)	kefir grains	CFS	*Aspergillus flavus*	[113]
fruit	LAB & other bacteria	*Lactobacillus lactis* subsp. *lactis* LABW1, LABW3, LABW4, *Burkholderia cenocepacia* VBC7 and *Pseudomonas poae* VBK1	jackfruit	rotten jackfruit	cells sprayed over the fruit	*Rhizopus stolonifer*	[114]
fruit	LAB	*Lactobacillus paracasei* ŁOCK0921 (1 tested in situ) (1/(9 in vitro/60))	wild cherries	plant and human	CFS cultivated with xylitol or galactosyl-xylitol directly on wild cherries	*Alternaria brassicicola* (1 tested in situ), *Alternaria alternata, Aspergillus niger*, *Fusarium lateritium*, *Geotrichum candidum*, and *Mucor hiemalis* (6/8)	[115]
fruit	LAB	Lactic acid bacteria strains LCM5, LAB 58, LAB 13, and LAB 43 (4/6)	apple	plants, fermented wheat bran, pickles, and sauerkraut	cells sprayed over the fruit (wounded and non-wounded)	*Penicillium expansum*	[116]
fruit/dairy	LAB	*Lactobacillus plantarum* TK9	citrus, apples and yogurt	Chinese naturally fermented congee	cells	*Penicillium roqueforti*, *Penicillium citrinum*, *Penicillium oxalicum*, *Aspergillus fumigatus*, *Aspergillus flavus*, and *Rhizopus nigricans (6/7)*	[117]
grain/seed	Other bacteria	*Acetobacter nigricans* AZT 54 (0/1)	maize, sorghum and wheat grains	paddy field soil samples	cereals submerged in suspension	*Fusarium sporotrichioides*, *Fusarium graminearum*, *Fusarium poae*, and *Fusarium equiseti* (4/10 in vitro)	[82]
grain/seed	Other bacteria	*Bacillus cereus*	peanut kernels	entomopathogenic nematode	cells and purified cyclo(4-hydroxy-l-Pro-l-Trp)	*Aspergillus flavus*	[118]
fruit	Other bacteria	*Bacillus subtilis* AFB22 [1/(50/200 in vitro)]	pomegranate	pomegranate leaves and fruits	cells and CFS by spraying on wounded fruits	*P. varsoniana* (only in situ), *A. flavus*, *A. clavatus*, *B. humicola, F. graminearum*, and *R. stolonifer* (in vitro)	[119]
fruit	Other bacteria	*Bacillus amyloliquefaciens* ZJ01 and ZJ02	jujube fruit	phyllosphere of Chinese jujube	whole culture in created wounds-prevention treatment	*Phoma destructiva* (2 strains)*, Alternaria alterna* (2 strains), and *Fusicoccum* spp. (5/5)	[120]
fruit	Other bacteria	*Bacilllus subtilis* V26	tomato fruit	rhizosphere of almond trees	whole culture, endospores, and CFS in created wounds	*Botrytis cinerea*	[84]
fruit	Other bacteria	*Paenibacillus polymyxa* APEC136 and *Bacillus subtilis* APEC170	apple	soil from several apple orchards	cells over created wounds	*Colletotrichum gloeosporioides*, *Colletotrichum acutatum*, and *Botryosphaeria dothidea*	[121]
fruit	Other bacteria	*Bacillus amyloliquefaciens* BUZ-14	apple, orange, grape, and cherries	surface of peach fruit from an orchard	cells, endospores and CFS	B*otrytis cinerea*, *Monilinia fructicola*, *Monilinia laxa*, *Penicillium digitatum*, *Penicillium expansum*, and *Penicillium italicum*	[122]
fruit	Other bacteria	*Cryptococcus laurentii*	peach fruit	surfaces of apple fruits	cells in created wounds	*Penicillium expansum*	[123]
fruit	Other bacteria	*Bacillus amyloliquefaciens* CPA-8	cherries	nectarine surface	wounded fruits packaged with in situ produced volatile organic compounds	*Monilia fructicola* (1/3)	[124]
fruit	Other bacteria	*Paenibacillus pasadenensis R16*	grape berries	leaf of grapevine plant	wounded fruit submerged in cell suspension	*Botrytis cinerea*	[125]
fruit	Yeast	*Cryptococcus laurentii* 2.3803	strawberries	not detailed	cells sprayed over fruits prior to harvest	*Botrytis cinerea*	[126]
fruit	Yeast	*Hanseniaspora uvarum*	grape berries	strawberries surface	cells in wounds and fruit submerged in salicylic acid or sodium bicarbonate	*Botrytis cinerea*	[127]
fruit	Yeast	*Wickerhamomyces anomalu*s BS91, *Metschnikowia pulcherrima* MPR3, A*ureobasidium pullulans* PI1, and *Saccharomyces cerevisiae* BCA62	grape berries	fermented olive brine and pomegranate	cells in created wounds	*Botrytis cinerea*	[71]
fruit	*Yeast*	*Aureobasidium pullulan* (25 strains)*, Cryptococcus magnus* (2 strains), *Candida sake* 2AM3 [(28/33 in situ) (33/55 in vitro)]	grape berries	surface of grape berries	wounded fruits submerged in cells suspension	*Aspergillus tubingensis*	[70]
fruit	Yeast	*Candida intermedia* and *Wickerhamomyces anomalus*	avocado	fruits, leaves, and the soil of the avocado orchards	cells in created wounds	*Colletotrichum gloeosporioides* and *Colletotrichum acutatum*	[128]
fruit	Yeast	*Hanseniaspora uvarum* Y3	orange	surfaces of grapes in vineyard	cells in created wounds	*Penicillium digitatum*	[85]
fruit	Yeast	*Pichia membranaefaciens*	citrus fruits *Citrus sinensis*	not detailed	cells in created wounds	*Colletotrichum gloerosporioides*	[129]
fruit	Yeast	*Rhodotorula minuta* ACBL-23, *Candida azyma* ACBL-44, S. cerevisiae ACBL-52, *Rhodotorula mucilaginosa* ACBL-68, and *Aureobasidium pullulans* ACBL-77	‘Pera’ orange fruits	citrus leaves, flowers, fruits, and citrus-growing soils	cells in created wounds	*Geotrichum citri-aurantii*	[130]
fruit	Yeast	*Pichia fermentans* (2 strains)*, Wickerhamomyces anomalus, Kazachstania exigua*, and *Saccharomyces cerevisiae*	lemons	surface of leaves and fruits of different citrus and wash-water from lemon shells	wounded fruits submerged cells suspension	*Penicillium digitatum* and *Penicillium italicum*	[131]
fruit	Yeast	*Debaryomyces hansenii* KI2a, *D. hansenii* MI1a, and *Wickerhamomyces anomalus* BS91	peach and plum fruits	blue-veined Rokpol cheese and fermented olive brine	cells in created wounds	*Monilinia fructigena* and *Monilinia fructicola*	[132]
fruit	Yeast	*Candida tropicalis* YZ27	banana	from bitter gourd	cells in created wounds	*Colletotrichum musae*	[133]
fruit	Yeast	*Yarrowia lipolytica*	grape berries	surface of grapes	cells in created wounds	*Talaromyces rugulosus*	[134]
meat	Yeast	*Debaromyces hansenii* FHSCC 253H	dry-cured ham slices	dry-cured meat products	cells over slices (a_w_ controlled)	*Penicillium nordicum*	[135]
meat	Yeast	*Debaromyces hansenii* 253H and 226G G	dry-fermented sausage	dry-cured meat products	cells over slices after fermentation	*Penicillium verrucosum*	[136]
meat	Molds	*Penicillium nalgoviense*	dry-fermented sausages	TEXEL PN1 from Danisco (Niebüll, Germany)	immersion of sausages in cells suspension	*Penicillium verrucosum*	[137]
meat	Molds	*Penicillium chrysogenum CECT 20922*	dry-cured ham slices	not detailed	cells	*Cladosporium cladosporioides*, *C. herbarum*, and *C. oxysporum*	[138]
fruit	Molds	*Clonostachys rosea*	tomato fruit	not detailed	cells sprayed over the fruit	*Botrytis cinerea*	[139]

**Table 2:** Examples of bioprotective cultures and fermentates used in processed food and postharvest fruits available in the market.

Product Name	Application Field	Properties	Composition	Manufacturer
Holdbac YM-B or YM-C	fermented food and white cheeses	protection against yeasts and molds	*Lactobacillus rhamnosus* and *Propionibacterium freudenreichii* subsp*. shermanii*	DuPont Danisco
Holdbac YM-XPM	fermented dairy and mild acidic yogurt	protection against yeasts and molds	*Lactobacillus plantarum*	DuPont Danisco
Holdbac YM-XPK	all types of cheeses	protection against yeasts and molds	*Lactobacillus plantarum*	DuPont Danisco
FreshQ 1 and FreshQ 4	cottage cheese	protection against yeasts and molds	*Lactobacillus rhamnosus* and *Lactobacillus paracasei*	CHR Hansen
FreshQ 2	cottage cheese	protection against yeasts and molds	*Lactobacillus rhamnosus*	CHR Hansen
FreshQ 5	cottage cheese	protection against yeasts and molds	*Lactobacillus paracasei*	CHR Hansen
Natamax	fruit juices, wine, surface of dry-ripened food, dairy, and bakery products	protection against yeasts and molds	Natamycin produced by *Streptomyces natalensis*	DuPont Danisco
MicroGard	sauces, salad dressings, prepared meals, cured meat, pastas, bakery and dairy products, hash brown potatoes	protection against Gram-positive bacteria, Gram-negative bacteria, yeasts, and molds	Fermentate *(skim milk or dextrose)* of *Propionibacterium freudenreichii* subsp*. shermani*	DuPont Danisco
Hi Shield P	bakery products, salad dressings, and general used in food industry	protection against molds, yeasts (*Pichia anomala*), and bacteria (*Bacillus subtilis*); increase sour taste; and reduce salt content (flavor improver and enhancer)	Fermentate (corn) of lactic acid bacteria and yeasts	HI-FOOD S.p.A.
Inhibit FOG, Inhibit 2800, Inhibit 1900CW, Inhibit 3600 and Inhibit 2100NF	bakery products, cheeses, meats, salad dressings, condiments, dips, spreads, and meats	protection against molds, yeasts, and Gram-negative bacteria	Fermentates (dextrose, wheat, wheat flour, whey, brown rice) of *Propionibacterium freudenreichii*	Mezzoni Foods
Biosafe 10LP	cherries, pome fruits, citrus, and potatoes	protection against *Penicillium expansum, Botrytis cinerea, Mucor piriformis, Fusarium sambucinum, Helminthosporium solani,* and *Rhizopus stolonifer*	*Pseudomonas syringae*	Nu Farm Inc. USA
Aspire	citrus and pome fruit	protection against molds (*P. expansum* and *Botrytis cinerea*)	*Candida oleophila*	Ecogen Inc. USA
Befresh	fresh fermented milk products	control the growth of yeast and molds	*Lactobacillus paracasei* and *Propionibacterium freudenreichii* subsp*. shermanii,*	Handary
Candifruit	pome fruit	protection against *Botrytis cinerea, Penicillium expansum*, and *Rhizopus stolonifer*	*Candida sake*	Sipcam-Inaagri, SA (Valencia, Spain)
Boni-Protect	pome fruit	protection against *Botrytis cinerea, Monilinia fructigena, Penicillium expansum*, and *Pezicula malicortici*	*Aureobasidium pullulan*	BioFerm GMbH, Germany
Shemer	citrus fruit, stone fruits, and berries	protection against *Aspergillus niger, Botrytis cinerea, Penicillium expansum, Penicillium digitatum, Penicilllium italicum*, and *Rhizopus stolonifer*	*Metschnikowia fructicola*	Bayer Cropscience, Israel
Pantovital	citrus and pome fruit	protection against *Botrytis cinerea, Penicillium expansum, Penicillium digitatum, Penicillium italicum*, and *Rhizopus stolonifer*	*Pantoea agglomerans*	BioDURCAL S.L.
YieldPlus	citrus, apple, and pear fruit	not detailed	*Cryptococcus albidus*	Anchor Bio-Technologies, Cape Town, South Africa
Nexy	pome fruit	protection against *Botrytis cinerea* and *Penicillium expansum*	*Candida oleophila*	BioNext sprl, France
Serenade	grapes, legumes, pome fruits, and peanuts	protection against fungi causing powdery mildew, late blight brown rot, fireblight	*Bacillus subtilis*	Agra Quees Inc.
